# Supplementary material for: Integrated Multi-Omics Investigations of Metalloproteinases in Colon Cancer: Focus on MMP2 and MMP9
Source: Int J Mol Sci. 2021 Nov 17;22(22):12389. doi: 10.3390/ijms222212389 (PMC8622288; doi:10.3390/ijms222212389)
Supplement: Supplementary file 1 [file ijms-22-12389-s001.zip › ijms-1431506-supplementary/Supplementary Figures IJMS.pptx]

## Slide 1
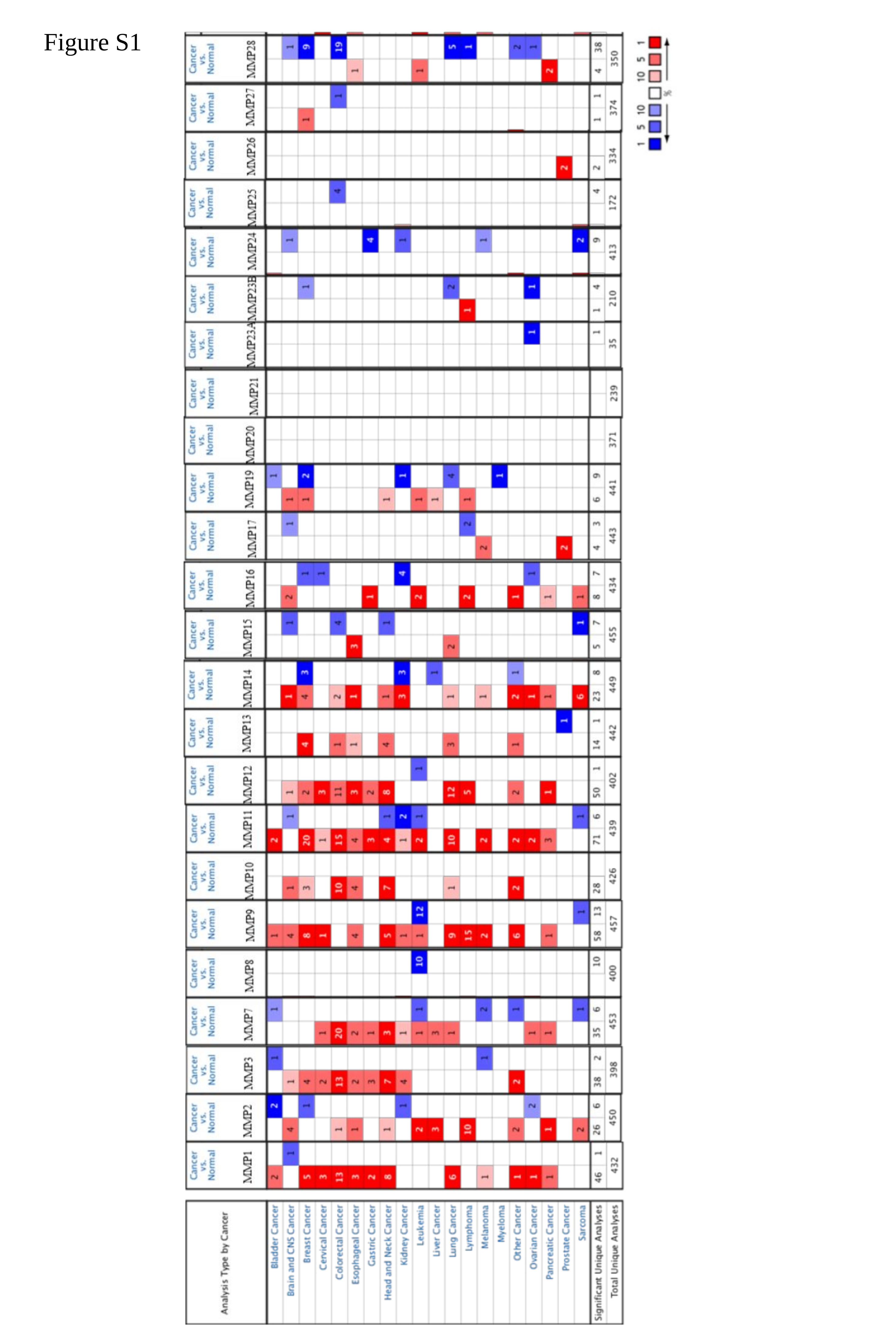

Figure S1

## Slide 2
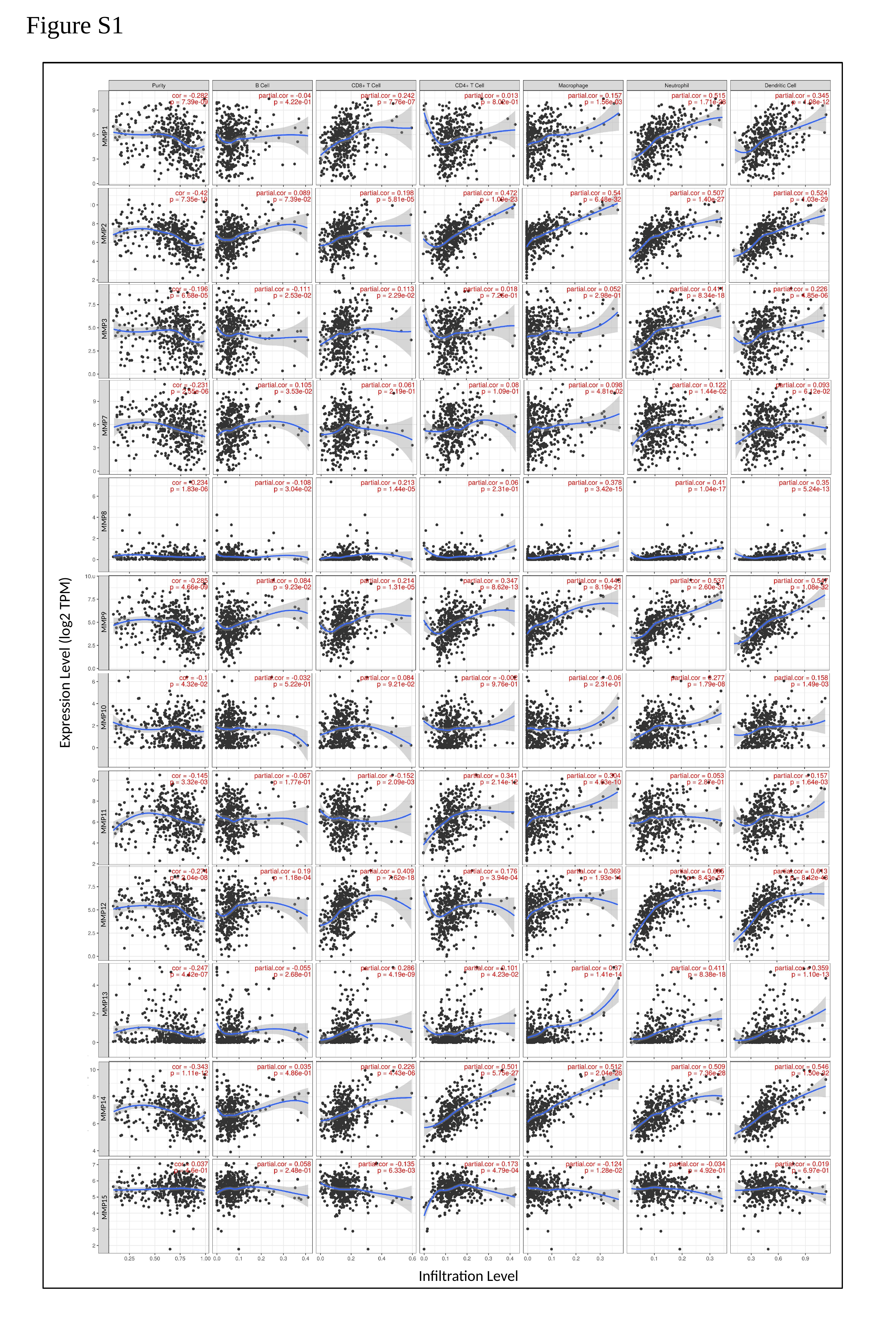

Figure S1
MMP1
MMP2
MMP3
MMP7
MMP8
MMP9
Expression Level (log2 TPM)
MMP10
MMP11
MMP12
MMP13
MMP14
MMP15
Infiltration Level

## Slide 3
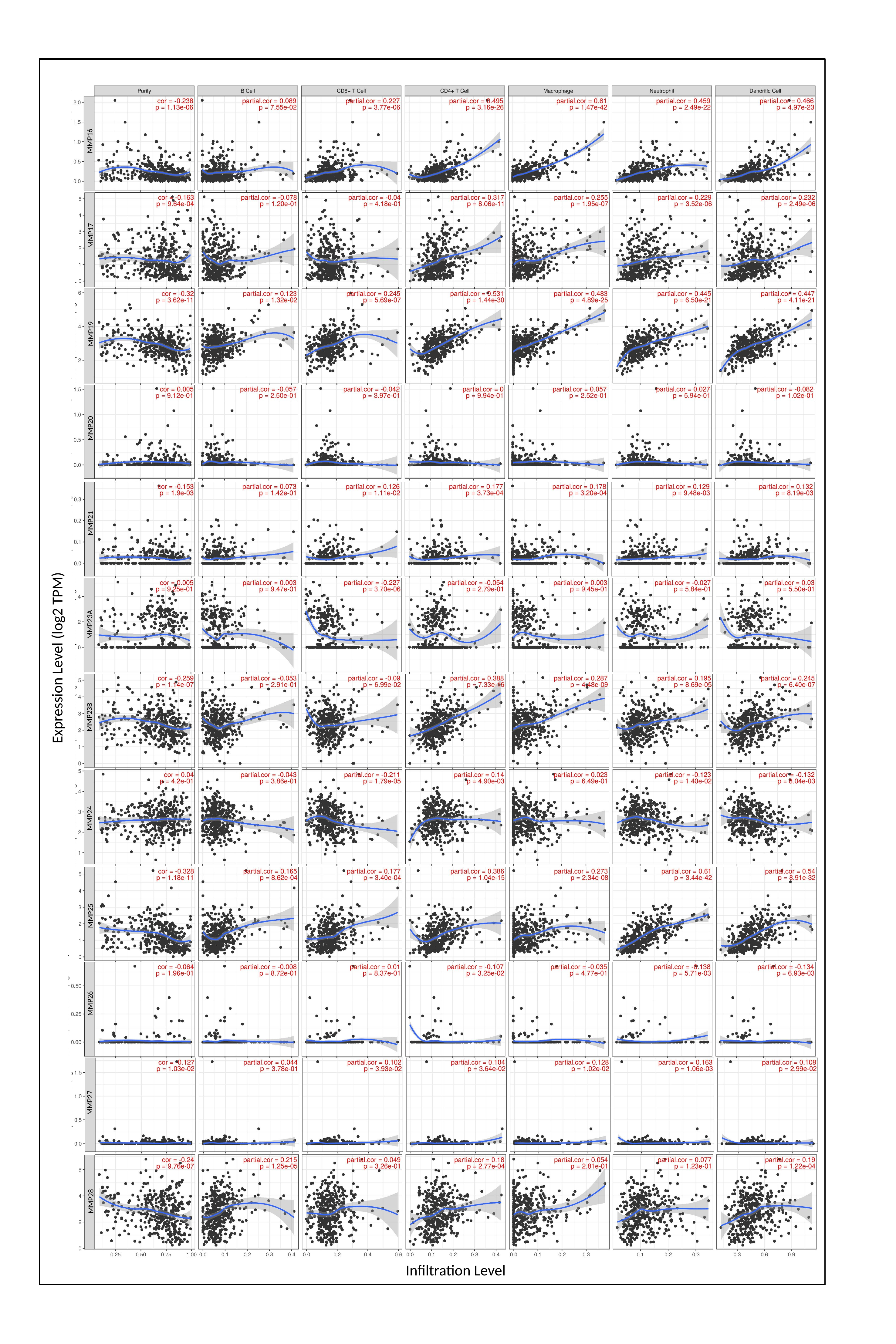

MMP16
MMP17
MMP19
MMP20
MMP21
MMP23A
Expression Level (log2 TPM)
MMP23B
MMP24
MMP25
MMP26
MMP27
MMP28
Infiltration Level
