# Supplementary figures and images for: Integrated Multi-Omics Investigations of Metalloproteinases in Colon Cancer: Focus on MMP2 and MMP9
Source: Int J Mol Sci. 2021 Nov 17;22(22):12389. doi: 10.3390/ijms222212389 (PMC8622288; doi:10.3390/ijms222212389)

## Slide 1
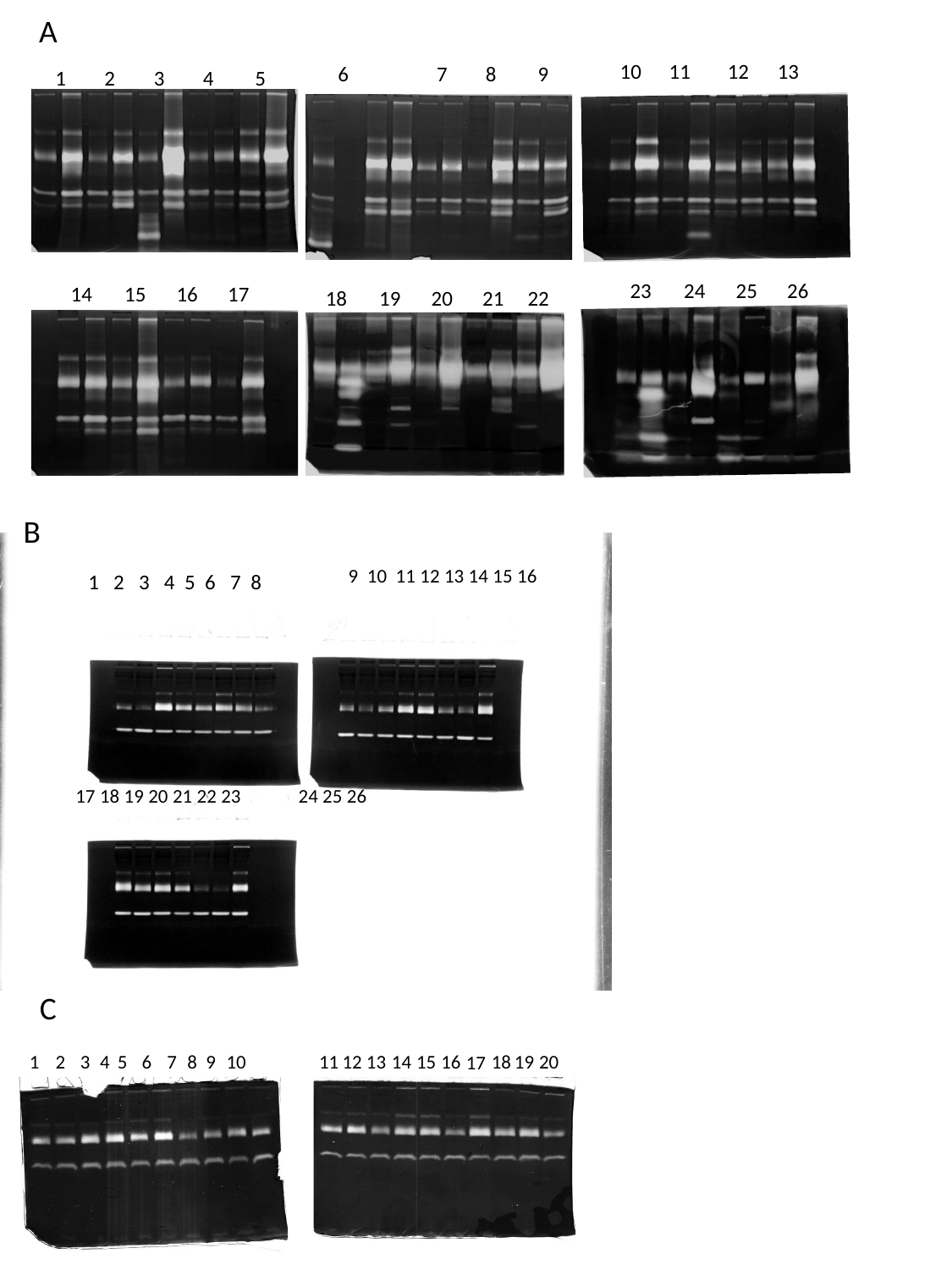

A
10
11
12
13
6
7
8
9
1
2
3
4
5
23
24
25
26
14
15
16
17
22
18
19
20
21
B
9 10 11 12 13 14 15 16
1 2 3 4 5 6 7 8
17 18 19 20 21 22 23 24 25 26
C
1
2
3
4
5
6
7
8
9
10
11
12
13
14
15
16
18
19
20
17

Supplement: Supplementary file 1 [file ijms-22-12389-s001.zip › ijms-1431506-supplementary/Original files from Figure 4.pptx]
